# Supplementary material for: Exploring the mechanism of hyperpermeability following glycocalyx degradation: Beyond the glycocalyx as a structural barrier
Source: PLoS One. 2021 Jun 4;16(6):e0252416. doi: 10.1371/journal.pone.0252416 (PMC8177458; doi:10.1371/journal.pone.0252416)
Supplement: Supplementary file 2 [file pone.0252416.s002.docx]

S1 Table

TranscriptID	GeneName	Type	GAG GAG	GAG GAG	LPS LPS2	WT WT1.0	WT WT2.0	GAG/WT
ENSMUST00000031319.7	Ppbp	protein_coding	15.31187	6.425698	1.241973	0.135599	0	160.3074
ENSMUST00000074898.7,ENSM	Hp	protein_coding	12.35604	15.12513	14.57285	0.457204	0	60.10697
ENSMUST00000167214.7,ENSM	Pla2g7	protein_coding	20.51661	15.41279	12.53226	0.606626	0	59.22826
ENSMUST00000032336.6,ENSM	Plbd1	protein_coding	13.5711	9.02611	2.111496	0.446195	0	50.64421
ENSMUST00000075072.5	Gpr65	protein_coding	10.86534	7.012529	10.835	0	0.457133	39.10864
ENSMUST00000064821.13,ENS	Itgam	protein_coding	11.17269	9.60463	10.31335	0.360412	0.21179	36.31112
ENSMUST00000098015.9,ENSM	RP23-144N15.4	protein_coding	7.695068	7.456466	7.391886	0.300557	0.221536	29.02079
ENSMUST00000021729.8,ENSM	Gpr132	protein_coding	8.918961	10.20435	2.477085	0.757258	0	25.25336
ENSMUST00000122088.1	S1pr5	protein_coding	7.077157	5.199578	1.219518	0.234966	0.283743	23.66786
ENSMUST00000069960.11,ENS	S100a9	protein_coding	18.74163	31.6937	21.81638	2.250636	0	22.40936
ENSMUST00000187516.6,ENSM	Slc11a1	protein_coding	7.584474	7.034694	4.25965	0.673824	0	21.69582
ENSMUST00000060484.8	Clec4a1	protein_coding	18.02512	11.79868	10.41743	1.602387	0	18.61211
ENSMUST00000093852.4,ENSM	Zbtb16	protein_coding	6.395979	6.337712	2.817584	0.694937	0.089514	16.23261
ENSMUST00000046091.6	Elane	protein_coding	5.147382	15.74562	0.546771	0.149242	1.201485	15.46797
ENSMUST00000028741.13,ENS	Haus2	protein_coding	6.36055	7.936088	4.932155	0.501987	0.440868	15.16313
ENSMUST00000043577.1	Cldn5	protein_coding	14.13194	13.27882	0.760994	0.519286	1.337778	14.76027
ENSMUST00000028846.6,ENSM	Dusp2	protein_coding	9.664556	18.91201	9.938515	1.97956	0	14.43582
ENSMUST00000170205.1	RP23-369M17.1	protein_coding	6.758018	8.720278	12.64745	1.114642	0	13.88634
ENSMUST00000019068.6,ENSM	Alox15	protein_coding	9.471721	6.420659	0.65773	0	1.156246	13.74481
ENSMUST00000025004.6	Adgre4	protein_coding	19.33845	12.91962	4.751944	2.566054	0	12.57108
ENSMUST00000054002.3,ENSM	Socs3	protein_coding	20.03997	21.57339	19.63383	2.031879	1.513098	11.73868
ENSMUST00000102858.9,ENSM	Atp1a3	protein_coding	6.835909	6.374495	1.512568	0.96128	0.277806	10.66141
ENSMUST00000115366.2	Slc12a2	protein_coding	8.555928	7.088723	1.787908	0.653185	0.942908	9.801842
ENSMUST00000098666.8,ENSM	Ceacam1	protein_coding	8.646098	7.798431	3.567668	1.100267	0.631245	9.497206
ENSMUST00000230931.1,ENSM	Nudt16l1	protein_coding	8.868886	8.402886	4.280983	1.884677	0	9.164315
ENSMUST00000207370.1,ENSM	Prmt1	protein_coding	7.049809	5.884651	8.80996	1.414944	0	9.141324
ENSMUST00000102802.9,ENSM	Btla	protein_coding	7.58387	5.059398	3.303794	1.397751	0	9.045438
ENSMUST00000209328.1,ENSM	Ifitm6	protein_coding	10.42675	5.513253	0.29725	1.784966	0	8.930144
ENSMUST00000030724.8	Sesn2	protein_coding	8.765332	9.691823	5.341522	2.070747	0.272175	7.877834
ENSMUST00000017637.12,ENS	Igfbp4	protein_coding	10.95122	15.92221	1.929052	1.967698	1.499244	7.751334
ENSMUST00000079970.5,ENSM	Hspbp1	protein_coding	8.948029	8.293153	8.862779	2.173315	0.124975	7.501743
ENSMUST00000041367.8,ENSM	Dcaf15	protein_coding	7.780825	8.967538	4.271823	1.547787	0.697793	7.458367
ENSMUST00000110042.2,ENSM	Sema4d	protein_coding	11.43899	9.592484	4.460753	1.72117	1.200891	7.197482
ENSMUST00000070518.3,ENSM	Nkg7	protein_coding	8.198534	6.823706	16.97264	0.321344	1.810904	7.045262
ENSMUST00000102521.8,ENSM	Rap1gap2	protein_coding	7.815857	6.891364	1.602153	2.090886	0	7.033965
ENSMUST00000033621.6,ENSM	Gla	protein_coding	13.19675	11.19992	16.12146	0.76463	2.751971	6.937571
ENSMUST00000036884.1	Klf9	protein_coding	15.90535	18.30911	15.41684	4.005519	0.965954	6.882157
ENSMUST00000177637.1,ENSM	Cx3cr1	protein_coding	8.116606	5.872908	0.716568	1.238724	0.839786	6.730548
ENSMUST00000016144.11,ENS	Plpp1	protein_coding	14.27326	12.68007	12.75883	3.608413	0.405347	6.715231
ENSMUST00000060989.8,ENSM	Sorl1	protein_coding	8.108373	8.402848	4.958371	2.360872	0.125317	6.641177
ENSMUST00000207955.1,ENSM	Tmem147	protein_coding	8.423404	9.977571	7.848586	2.773322	0	6.634993
ENSMUST00000151243.1,ENSM	Hfe	protein_coding	10.87765	9.2816	3.163068	2.384528	0.66196	6.61721
ENSMUST00000111222.1,ENSM	Ldlrad3	protein_coding	7.073171	5.373744	1.831225	1.926194	0	6.461921
ENSMUST00000055770.3	Hist1h1a	protein_coding	17.50984	27.37683	54.09271	0.594551	6.381977	6.433955
ENSMUST00000131208.1,ENSM	1-Sep	protein_coding	6.051943	7.223299	5.549638	1.860257	0.256734	6.270806
ENSMUST00000020308.4	Ddit4	protein_coding	11.03026	14.06545	4.036449	2.00826	2.02096	6.228429
ENSMUST00000100239.2	RP23-331L12.7	protein_coding	9.94421	11.94976	1.642906	2.071814	1.448686	6.218994
ENSMUST00000166939.7,ENSM	Adgre5	protein_coding	54.36026	49.21658	20.9438	9.185705	7.520906	6.199752
ENSMUST00000019266.5,ENSM	Ccl9	protein_coding	27.95294	22.71382	222.4461	2.893675	5.319205	6.169182
ENSMUST00000031264.11,ENS	Plac8	protein_coding	17.38956	17.32005	8.3362	5.626944	0	6.168464
ENSMUST00000173393.7,ENSM	Rbm38	protein_coding	5.586504	7.253901	3.125496	2.098646	0	6.118424
ENSMUST00000041190.16,ENS	Mcrs1	protein_coding	6.411974	5.458453	4.55258	1.884892	0.067442	6.080121
ENSMUST00000031320.7,ENSM	Pf4	protein_coding	8.75719	6.647074	2.611761	0	2.623603	5.871415
ENSMUST00000025278.7,ENSM	Mrpl27	protein_coding	12.15099	9.397791	13.83747	2.981808	0.720159	5.8209
ENSMUST00000001963.13,ENS	Ace	protein_coding	64.09513	67.29655	9.209583	10.6106	12.43309	5.701852
ENSMUST00000163326.7,ENSM	Atp2a3	protein_coding	13.75591	14.66928	6.452335	3.309151	1.701418	5.673045
ENSMUST00000096318.3	Ap5b1	protein_coding	7.292938	7.580043	5.104141	2.137267	0.535306	5.565041
ENSMUST00000104959.1	RP23-14F5.5	protein_coding	13.2322	12.7629	16.49663	4.801342	0	5.414132
ENSMUST00000162726.4	Znhit2	protein_coding	9.695329	5.534999	4.620024	2.866004	0	5.314134
ENSMUST00000086040.5	F5	protein_coding	11.78499	8.102582	0.965496	2.790226	1.012958	5.22919
ENSMUST00000087315.13,ENS	Vars	protein_coding	13.20125	14.38755	15.92578	4.058911	1.433555	5.023026
ENSMUST00000001046.6,ENSM	S100a4	protein_coding	11.46498	8.712279	3.301225	0	4.04884	4.983465
ENSMUST00000174064.8,ENSM	Apoe	protein_coding	109.1385	84.35171	14.59557	18.75171	21.45544	4.812332
ENSMUST00000039803.6,ENSM	Ubac2	protein_coding	7.397126	11.93659	7.225513	2.973491	1.074783	4.775792
ENSMUST00000126621.2	RP23-366P9.8	protein_coding	5.945784	7.766314	7.92042	2.31631	0.55943	4.768199
ENSMUST00000094451.3	Gpr157	protein_coding	5.720631	5.627249	2.516263	0.929076	1.495922	4.67954
ENSMUST00000102541.9,ENSM	Gale	protein_coding	5.474594	6.121909	2.95118	2.478549	0	4.678747
ENSMUST00000102899.9,ENSM	Med22	protein_coding	12.36393	14.36916	9.292084	3.494589	2.224509	4.674352
ENSMUST00000055984.6	Isg20l2	protein_coding	11.12828	12.8897	13.77758	4.873137	0.32693	4.618783
ENSMUST00000092459.3	Cd300lh	protein_coding	12.70735	15.99055	1.301906	1.749493	4.468171	4.615543
ENSMUST00000062787.7	Cd300e	protein_coding	10.10187	8.14352	3.092085	2.391301	1.585408	4.588063
ENSMUST00000095218.3	Rpl7a-ps5	protein_coding	11.08251	11.45439	13.70169	2.243755	2.675104	4.581733
ENSMUST00000003635.6	Ier3	protein_coding	8.343245	22.40617	43.28919	3.375955	3.345036	4.575131
ENSMUST00000075558.4	Hist1h3f	protein_coding	18.03486	27.86247	31.21258	9.578702	0.485655	4.560383
ENSMUST00000128694.7,ENSM	Chka	protein_coding	8.055549	10.94187	12.52135	2.613669	1.553537	4.558791
ENSMUST00000103232.1	RP23-406N5.7	protein_coding	20.57889	26.65955	17.76336	6.022574	4.502203	4.488307
ENSMUST00000036572.3	Tmem51	protein_coding	7.95602	9.943974	4.399831	3.994429	0	4.481239
ENSMUST00000229582.1	Tmem51	protein_coding	7.95602	9.943974	4.399831	3.994429	0	4.481239
ENSMUST00000174699.7,ENSM	H2-Q6	protein_coding	21.72365	22.13124	23.71128	3.354047	6.520866	4.44104
ENSMUST00000208740.1,ENSM	Hcst	protein_coding	7.207468	9.638547	3.850117	1.83059	1.964977	4.438339
ENSMUST00000049474.8,ENSM	Fbxw8	protein_coding	6.429783	7.058251	5.128664	1.647955	1.412294	4.407496
ENSMUST00000031341.10,ENS	Cdk2ap1	protein_coding	9.343453	9.372836	13.46352	4.139898	0.113788	4.400016
ENSMUST00000022586.1	Nufip1	protein_coding	6.110811	7.169763	7.152879	2.438945	0.594998	4.377332
ENSMUST00000096243.5	B3gat3	protein_coding	10.40108	13.73714	9.969609	3.898374	1.624594	4.370517
ENSMUST00000003906.12,ENS	Farsa	protein_coding	8.921442	8.7453	7.834804	4.109842	0	4.298643
ENSMUST00000004774.3	Aqp1	protein_coding	10.0365	11.54582	0.887489	3.633617	1.440136	4.253719
ENSMUST00000098571.4	AC155163.2	protein_coding	11.45171	8.650302	5.223523	4.073621	0.655901	4.250327
ENSMUST00000157027.7,ENSM	Eno3	protein_coding	7.461063	6.606651	2.371962	2.364527	0.951792	4.241967
ENSMUST00000172633.7,ENSM	H2-T22	protein_coding	10.12062	11.77703	9.091546	2.601562	2.562605	4.240307
ENSMUST00000107330.2	RP23-326D6.5	protein_coding	10.96763	11.26081	3.317405	0	5.30162	4.192763
