# Supplementary material for: Exploring the mechanism of hyperpermeability following glycocalyx degradation: Beyond the glycocalyx as a structural barrier
Source: PLoS One. 2021 Jun 4;16(6):e0252416. doi: 10.1371/journal.pone.0252416 (PMC8177458; doi:10.1371/journal.pone.0252416)
Supplement: Attachment — Submitted filename: Response to Reviewers0513.docx [file pone.0252416.s004.docx]

**Response to reviewers regarding the manuscript by Kyoko Abe et al.**

Thank you for taking the time to review our work and provide constructive feedback. In the manuscript, all changes have been highlighted with “Track changes” option in Microsoft Word.

**Reviewers' comments:**

**Reviewer #1:**
Minor comments:
***1. Page 6, line 66, "One hour after the injection of the enzymes,..." and line 71, "Twenty-four hours after the first LPS injection,...". An proof of time chosen here is needed, it can be a citation from previous study or a time dependence test.***

**Response:** Thank you for your suggestion. We have cited the following references and added them in page 6, on lines 67 and 72 of the revised manuscript.

References

1. Uhl B, Hirn S, Immler R, Mildner K, Möckl L, Sperandio M, et al. The Endothelial Glycocalyx Controls Interactions of Quantum Dots with the Endothelium and Their Translocation across the Blood-Tissue Border. ACS Nano. 2017;11(2):1498-1508.

2. Kataoka H, Ushiyama A, Akimoto Y, Matsubara S, Kawakami H, Iijima T. Structural Behavior of the Endothelial Glycocalyx Is Associated with Pathophysiologic Status in Septic Mice: An Integrated Approach to Analyzing the Behavior and Function of the Glycocalyx Using Both Electron and Fluorescence Intravital Microscopy. Anesth Analg. 2017;125(3):874-883.

***2. Page 13, Fig. 1 needs to indicate how many instances are included in this figure.***

**Response:** Thank you for your suggestion. We apologize for the lack of this important infomation. Actually, TEM images from one out of three mice for each group were shown in Fig.1 A, B, C. The precise number of replicate is now shown on page 13, line 189-192 of the revised manuscript.

***3. Page 15, line 217. "The arrows. indicate the FITC-labeled dextran...", there is no arrow in Fig. 2.***

**Response:** Thank your for your suggestion. We apologize for this mistake. We have added the arrows in Fig. 2 of the revised manuscript.

**Major comments:**

***1. Page 14, "Furthermore, FITC-labeled dextran was not exuded from the venules in enzyme-injected mice, ... LPS-injected septic mice (Fig. 2D)." In this section, firstly no difference was mentioned between Fig. 2B and C, then another statement of no significant difference between Fig. 2A and C was made. It is confusing. A clear statement of comparing among Fig. 2 A-C should be made. In addition to this, there is no statement indicates whether Fig. 2A-C is based only on one instance or averaged result. If it is based on only one replica, a statistically analysis is needed.***

**Response:** Thank you for your indication. We apologize for this confusion. Therefore, we corrected this statement as follows: The exudation of FITC-labeled dextran was not found in control and enzyme-injected mice, while the exudation of FITC-labeled dextran was found in LPS-injected mice (Fig. 2A-C, n=7 for control; n=7 for GAGs-digestion model; n=7 for septic model). We have added this in page 14, on lines 201-204 of the revised manuscript.

In addition, ｗe also appreciate an another comment regarding to Fig. 2A-C. Actually, in Fig.2A-C, representative images of fluorescent intensity from one out of 7 mice for each condition was shown. In addition, in Fig.2D, we presented the averaged value of the intensity in each group. We would add the number of replication and P-value as follows: *P* = 5.257e-05, compared with the septic model, n=7 for control; n=7 for GAGs-digestion model; n=7 for septic models. We have added this in pages 14-15, on lines 207-208, 216, 220, 222 of the revised manuscript and in the figure legends.

***2. Page 15, "The mean intensity of the GAGs-digested HUVECs was also significantly ... endothelial cells in vitro." In this section, "significant" was used to show the difference in Fig. 3B and "no significant difference" to show Fig. 3C. A more clear definition is needed for how much can be called "significant". A quantitive result is helpful. Moreover, in Fig. 3C, as time goes, the differences become larger (12 mins) than short time result (3 mins). A time dependent result change should be made for a solid conclusion. And an underline relationship explanation among panels in Fig. 3 is needed.***

**Response:** Thank you for your suggestions. In Fig.3B, We presented the averaged value of the intensity obtained from three independent experiments. The number of replication and exact P-value as follows: *P* = 0.029212854, n=3 for control; n=3 for GAGs digestion. We have added this in page 16, on lines 238-239 of the revised manuscript.

As the reviewer mentioned regarding Fig. 3C, it appears that the differences become larger (12 mins) than short time result (3 mins) in GAGs digestion compared with control. However, there was no statistically significant difference between them even after 12 mins (*P*=0.4013130). Therefore, we have revised the sentences as follows: No statistically significant difference in the TEER was seen between HUVECs with and those without digestion (Fig 3C, n=3 for control; n=3 for GAGs digestion). The HUVECs with or without GAGs digestion exhibited a rapid 10% decrease in the TEER after 2 minutes the addition of histamine (3×10-6 mol/L), compared with untreated HUVECs (Fig. 3C, P = 0.0007866, n=3 for control; n=3 for control+Histamine; n=3 for GAGs digestion+Histamine), while no significant difference in the TEER after the addition of histamine was seen between HUVECs with and those without digestion (n=3 for control+Histamine; n=3 for GAGs digestion+Histamine).

The text is now shown in on page 16, line 241-248 of the revised manuscript.

***3. Page 23, "Our negative results may have arisen from the use of a different cell line." Either a prof of chosen of current cell line or a quantity analysis of result change based on varying cell lines is needed.***

**Response:** We apologize for our misunderstanding the cited paper (Singh A, J Am Soc Nephrol 18: 2885–2893, 2007; we have revised the reference list.). The paper reported that glycocalyx digestion by heparinase III did not affect on TEER of a glomerular endothelial cell line, while increased the albumin passage across the monolayers. Therefore, this finding is consistent with our result despite using different cell lines. However, we did not examine the albumin passage. We would like to examine this in the future studies. We have added the sentence about this to the text in pages 23 lines 353-357 of the revised manuscript.

**Reviewer #2:**

***These investigations attempt to evaluate the changes in vascular permeability and gene expression in endothelial barriers in mouse lung and cultured HUVECs when the glycocalyx is removed under conditions of sepsis and enzyme degradation and histamine. Unfortunately the experimental conditions are not adequate to effectively examine these questions. In the permeability experiments the authors do not take into account that the glycocalyx and the endothelial cells lie in series so the properties of both barriers must be taken into account when interpreting the results. Thus the use of the very large dextran (MW 2000KD) is restricted by the endothelial barrier with intact junction which is likely to be the case when only enzyme degradation is investigated. The observation that there is no increased leakage of the dextran when the glycocalyx is removed tells nothing about the contribution of the glycocalyx to the normal barrier. On the other hand the sepsis conditions modify both glycocalyx and the endothelial integrity resulting in breakdown of both barriers.
The use of TEER are also a problem. The glycocalyx offers very little resistance to small ions. Most of the small reported resistance is due to the reduced area for transport through the inter-endothelial junctions. Thus the failure to see changes in TEER with enzyme degradation is the expected result and provides no information about the status of the junctions On the other hand the fall in resistance after histamine is exactly as expected when histamine transiently opens the junctions between endothelial cells.
Thus the permeability investigating need to be replaced by studies that use a lower molecular weight dextran to investigate large molecule permeability in both in vivo and invitro experiments.
The gene expression are potentially more interesting but again the experimental design limits the usefulness of the result from enzyme degradation. Specifically expression in mouse lung of enzyme digested glycocalyx was examined after only 1 hour after the digestion. The cells from the septic model had been exposed to inflammatory conditions for 24 hours. The observation that these there was no change in genes associated with endothelial leakage (VE cadherin, PECAM1) were not differentially expressed over this period provides cannot be used to conclude that removal of the glycocalyx has no effect on gene regulating permeability. However the observation that genes associated with the cytokine mediated response are upregulated is of interest . However it is not clear where this is a direct result of glycocalyx removal, or activation of a range of other vascular cells which themselves interact with endothelial when their own glycocalyx is modified. Further control studies are required to distinguish some of these complex interactions.***

**Response:**

As the reviewer suggested, the use of the very large dextran (MW 2000KD) is possibly restricted by the endothelial barrier with intact junction after only enzyme degradation of the glycocalyx. However, in this study, we would like to clarify whether exposed receptors on the endothelial cells by enzymatic degradation of glycocalyx could induce paracellular permeability of endothelial cells for comparatively large molecules because various receptors on the endothelial cells are known to be buried in the glycocalyx. As our result, against our expectation, paracellular permeability for large dextran did not change after enzymatic digestion of glycocalyx *in vivo*. As the reviewer pointed out, the glycocalyx offers very little resistance to small ions but not large molecules. However, we would like to focus on the important point that TEER was not changed by histamine after enzymatic digestion of glycocalyx *in vitro*, suggesting that only exposure of receptor by glycocalyx degradation might not affect permeability for at least paracellular level. However, we cannot deny this possibility without investigating the effects of other ligands besides histamine, which may regulate the permeability. In this relation, to examine whether enzymatic degradation of glycocalyx can induce receptor-mediated activation of signal transduction pathways on endothelial cells, RNA sequencing was conducted one hour after the enzyme digestion. Gene ontology analysis revealed that the cytokine-mediated signaling pathway was upregulated. Indeed, as the reviewer suggested, it is not clear whether this is a direct result of glycocalyx removal, or activation of a range of other vascular cells which interact with endothelial when their own glycocalyx is modified. We would like to verify these important points in the future studies.

Here, we would like to emphasize that this is the first comprehensive analysis *in vivo* to examine the alteration of gene expressions of endothelial cells by the only enzymatic degradation of the glycocalyx.

We have added this point in the discussion of the revised version in page23, lines 357-366.
